# Risk factors that limit use of oral JAK inhibitors in chronic hand eczema: Findings from the Danish Skin Cohort

**DOI:** 10.1016/j.jdin.2024.08.006

**Published:** 2024-09-02

**Authors:** Yuki M.F. Andersen, Johan Sieborg, Lea Nymand, Tiago Torres, Andrea Chiricozzi, Simon Francis Thomsen, Jacob P. Thyssen, Alexander Egeberg

**Affiliations:** a Department of Dermatology and Venereology, Bispebjerg Hospital, Copenhagen, Denmark; b Department of Dermatology, Centro Hospitalar Universitário do Porto, Instituto de Ciências Biomédicas Abel Salazar, University of Porto, Porto, Portugal; c Dermatologia, Dipartimento Universitario di Medicina e Chirurgia Traslazionale, Università Cattolica Del Sacro Cuore, Rome, Italy; d Dermatologia, Dipartimento di Scienze Mediche e Chirurgiche, Fondazione Policlinico Universitario A. Gemelli IRCCS, Rome, Italy; e Department of Biomedical Sciences, Faculty of Health and Medical Sciences, University of Copenhagen, Copenhagen, Denmark; f Department of Clinical Medicine, Faculty of Health and Medical Sciences, University of Copenhagen, Copenhagen, Denmark

**Keywords:** chronic hand eczema, Danish Skin Cohort, JAK inhibitors, risk factors

## Abstract

**Background:**

Oral Janus kinase inhibitors (JAKis) have black-box warnings of infections, cancer risk, and cardiovascular and venous thromboembolic events. They may be used off-label for chronic hand eczema (CHE).

**Objectives:**

Assess the prevalence of risk factors potentially impacting oral JAKi safety in CHE patients.

**Methods:**

In the Danish Skin Cohort, CHE patients were examined for risk factors affecting oral JAKi use at baseline and followed for 12 months. Data were collected through register linkage (eg, cancer history) and through patient interviews (eg, smoking habits).

**Results:**

Of 941 adults with CHE (66.2% women; mean age 55.5 [SD 13.3] years), 768 (81.6%) patients had at least one risk factor potentially impacting oral JAKi use, of which 682 (72.5%) had nonmodifiable risk factors. Most common risk factors were current or former heavy smoking (62.8%, *n* = 591), obesity (28.1%, *n* = 264), hypercholesterolemia (21.5%, *n* = 202), and hypertension (18.8%, *n* = 177). Among patients without any risk factors at baseline (*n* = 173), 20.2% (*n* = 35) developed ≥1 risk factor during the following 12 months.

**Limitations:**

Certain risk factors may be underreported.

**Conclusion:**

Most CHE patients have risk factors limiting appropriateness of oral JAKi use. Health care providers should assess risk factors in their patients when choosing treatment for CHE.

**Capsule Summary**

• Oral Janus kinase inhibitors have black box warnings due to increased risk of cardiovascular events, serious infections, and certain cancers.
• More than eight out of every ten patients with chronic hand eczema have at least one risk factor impacting the use of oral Janus kinase inhibitors. Among patients without any risk factors, 20% developed one or more risk factors during a 12-month follow-up period.

## Introduction

Hand eczema is a common inflammatory skin condition affecting upward of 10% of adults during the course of one year^1^^,^^2^ and some may develop chronic hand eczema (CHE), defined as eczema on the wrists and/or hands that persists for at least 3 months or that relapses ≥2 times in a year.^3^ Standard of care for hand eczema includes avoidance of trigger factors and daily emollient use.^3^ Treatment with topical corticosteroids is often used both in acute and prolonged periods, although topical corticosteroids may be ineffective for irritant contact dermatitis and may cause atrophy with continued use.^4^ For some patients, systemic treatment is needed, and while alitretinoin, an oral retinoid, is the only currently approved systemic treatment (Europe and Canada) for severe CHE, oral and topical Janus kinase inhibitors (JAKis) are being tested for some or all subtypes of CHE in clinical trials.^5^^,^^6^

In recent years, safety concerns of oral JAKi have been raised by regulatory authorities including the US Food and Drug Administration and European Medicines Agency (EMA). Notably, labels of drugs such as tofacitinib, abrocitinib, baricitinib, and upadacitinib now have black box warnings that include risks of serious heart-related events, cancer, blood clots, and death.^5^ Moreover, in 2023 EMA restricted the use of most oral JAKis in patients aged ≥65 years, in current or previous long-term smokers, and patients with increased risk of cancer or major cardiovascular problems.^7^ Importantly, a recent study found that half of adults with atopic dermatitis (AD) had risk factors that could impact the use of oral JAKi due to potential safety concerns.^8^

With the outlook of oral JAKis being used in patients with CHE,^6^^,^^9^ with or without concurrent AD, a thorough understanding of the patient profile and risk factor composition among adults with CHE is needed. We therefore assessed the prevalence and development of risk factors impacting oral JAKi appropriateness in population-based cohort of adult patients with CHE in Denmark.

## Materials and methods

The study was registered at the Capital Region’s inventory (Videncenter for Dataanmeldelser, ref. P-2021-386), and informed written consent was obtained from all participants prior to study initiation. This constitutes the necessary legal requirements, as ethical approval is not needed for this type of study in Denmark.

The Danish Skin Cohort is a nationwide dermatologist-led prospective cohort established in 2018, comprising Danish adults (age ≥18 years) with skin diseases. In January 2022, the cohort was expanded to include patients with CHE. Patients are identified from visits to academic hospital centers as well as from a number of private practice dermatology clinics in Denmark. The Danish Skin Cohort, including design, scope, and data collection methods, has previously been described in detail.^10^ Briefly, patients with dermatologist-verified CHE in the Danish Skin Cohort were interviewed in a structured manner through a secure digital system, with clinical photographs being made available as appropriate. Patients’ severity of CHE was defined based on the photographic guide by Coenraads and colleagues.^11^

From January 14, 2022 through February 17, 2022 (“baseline assessment”), patients with CHE from the Danish Skin Cohort reported data regarding specific risk factors such as smoking history, height and body weight (enabling calculation of body mass index). This information was linked with nationwide registries, including the Danish National Patient Registry^12^ and the National Prescription Registry,^13^ allowing for complete information on comorbidity and pharmacotherapy use for all patients.

Patients were assessed at baseline and again 12 months later (interview period between January 3rd 2023 and January 31st 2023) to capture new risk factors developing since baseline.

In line with the EMA recommendations, risk factors included diabetes, hypertension, hypercholesterolemia, current or former long-term smoking (≥10 pack-years), obesity (body mass index ≥30 kg/m^2^), history of cancer (excluding keratinocyte cancer), venous thromboembolism (VTE), major adverse cardiovascular events (MACE), use of hormonal contraceptives in the past 12 months, and age ≥65 years, respectively. Hormonal contraceptives only included drug classes associated with increased VTE risk.^14^

We categorized risk factors as “nonmodifiable” (ie, long-term smoking, history of cancer, VTE, MACE, or age ≥65 years) and “modifiable” (conditions where the risk assessment could be impacted, eg, by effective treatment, ie, conditions including diabetes, hypertension, hypercholesterolemia, obesity, and hormonal contraception use in the previous year). Modifiable risk factors were included if they were present (eg, a recorded diagnosis of diabetes) within the last 12 months prior to baseline, whereas nonmodifiable risk factors (eg, previous cancer) were included if they occurred any time between January 1st, 1995 (registry inception) and baseline.

### Statistical analysis

Summary statistics were created and presented as frequencies with percentages for categorical variables and means with SDs or medians with interquartile ranges (IQRs), as appropriate, for continuous variables. Analyses were performed using Python v. 3.7.4 (Python Software Foundation).

## Results

The study comprised a total of 941 adults with CHE, including 623 (66.2%) women. Mean age at baseline was 55.5 (SD 13.3) years (Table I). Approximately one-third of patients (30.9%, *n* = 291) had a history of AD, with 174 (18.5%) and 254 (27.0%) patients having asthma and allergic rhinitis, respectively.

**Table I:** Characteristics of the study population

	Chronic hand eczema
	(*n* = 941)
Age, mean (SD)	55.5 (13.3)
Sex, *n* (%)	
Female	623.0 (66.2)
Male	318 (33.8)
History of AD, *n* (%)	291 (30.9)
History of asthma, *n* (%)	174 (18.5)
History of allergic rhinitis, *n* (%)	254 (27.0)
Current CHE severity, *n* (%)	
Clear or almost clear	731 (77.7)
Moderate	163 (17.3)
Severe	39 (4.1)
Very severe	8 (0.9)
DLQI, mean (SD)	3.5 (4.0)
Skin pain (NRS 0-10), mean (SD)	1.7 (2.4)
Pruritus (NRS 0-10), mean (SD)	2.9 (2.8)

*AD*, Atopic dermatitis; *CHE*, chronic hand eczema; *DLQI*, dermatology life quality index; *NRS*, numerical rating scale.

### Risk factors limiting use of oral JAKi

The mean number of risk factors was 1.8 (SD 1.5) and was higher for men (mean 2.2, SD 1.7) than women (mean 1.7, SD 1.3). More than eight of every ten patients (81.7%, *n* = 769) had at least one risk factor, including 682 (72.5%) patients with ≥1 nonmodifiable risk factors, leaving only 18.3% (*n* = 172) without any risk factors impacting oral JAKi use (Table II). Stratified by sex, 123 (19.7%) women and 49 (15.4%) men did not have any risk factors. Overall, 29.9% (*n* = 281) had one risk factor (women = 201 [32.3%]; men = 80 [25.2%]), and 51.9% (*n* = 488) carried two or more risk factors (women = 299 [48.0%]; men = 189 [59.4%]).

**Table II:** Prevalence of risk factors limiting the use of oral JAK inhibitors

	Chronic hand eczema
	(*n* = 941)
Number of risk factors, *n* (%)	
No risk factors	173 (18.4)
One risk factor	280 (29.8)
Two or more risk factors	488 (51.9)
Number of nonmodifiable risk factors, *n* (%)	
No nonmodifiable risk factors	259 (27.5)
One nonmodifiable risk factor	427 (45.4)
Two or more nonmodifiable risk factors	255 (27.1)
Specific risk factors, *n* (%)	
Diabetes, *n* (%)	69 (7.3)
Hypertension, *n* (%)	177 (18.8)
Hypercholesterolemia, *n* (%)	202 (21.5)
Current or former long-term smoking∗, *n* (%)	591 (62.8)
Obesity, *n* (%)	264 (28.1)
Hormonal contraception, *n* (%)	18 (1.9)
Cancer ex NMSC, *n* (%)	74 (7.9)
VTE, *n* (%)	22 (2.3)
MACE, *n* (%)	37 (3.9)
Age ≥ 65, *n* (%)	257 (27.3)

*CHE*, Chronic hand eczema; *DLQI*, dermatology life quality index; *JAK*, Janus kinase; *MACE*, major adverse cardiovascular event; *NMSC*, nonmelanoma skin cancer; *VTE*, venous thromboembolism.

∗ Defined as ≥10 pack years.

The most common risk factors were current or former heavy smoking (62.8%, *n* = 591), obesity (28.1%, *n* = 264), hypercholesterolemia (21.5%, *n* = 202), hypertension (18.8%, *n* = 177), and diabetes (7.3%, *n* = 69). Hormonal contraceptives were used by 1.9% (*n* = 18). A total of 74 (7.9%) patients had a history of cancer (excluding keratinocyte cancer), and 3.9% (*n* = 37) and 2.3% (*n* = 22) patients had a history of MACE and VTE, respectively.

### Risk factors by age groups

Stratified by current age (18-49, 50-64, ≥65, respectively), there were a total of 283, 396, and 257 patients with CHE, respectively. The prevalence of most risk factors increased with ascending age groups (eg, hypertension: 2.8%, 20.2%, and 34.6%); however, obesity was most common in the age group 50-64, affecting 30.1%, while 28.3% of the age group 18-49 and 25.3% of ≥65 age group were obese. Hormonal contraceptives were only seen in the 18-49 group, as expected.

### Risk factors according to current CHE severity

A total of 210 patients currently had moderate to severe CHE. Patients with moderate to severe CHE had a slightly higher prevalence of carrying one or more risk factors for oral JAKi use (85.7% vs 80.6%). Among the measured risk factors, smoking and diabetes were more prevalent (67.1% vs 61.6% and 10.5% vs 6.4%) when compared to patients with mild CHE.

### Development of risk factors during 12-month follow-up

**Fig 1:**
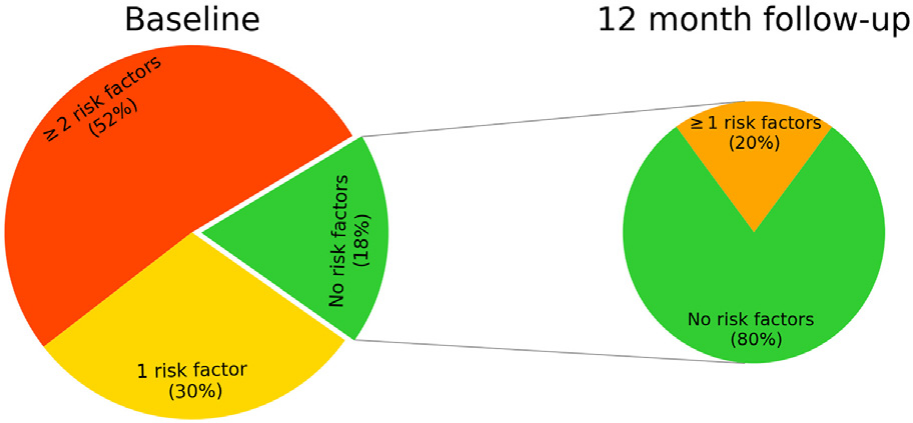
Proportion of patients reporting with no, 1, or ≥2 risk factors at baseline and distribution at 12-month follow-up among patients without risk factors at baseline.

Among the patients (*n* = 173) without any risk factors at baseline, 20.2% (*n* = 35) had developed at least one risk factor at follow-up 12 months later, of which 29 were nonmodifiable (Fig 1). The most frequent risk factor developed was smoking, followed by hypertension.

## Discussion

In this population-based study of 941 adults with dermatologist-verified CHE, we found that 81.7% of patients had at least one risk factor that would limit the use of oral JAKi. Importantly, among patients without any such risk factors, 20.2% developed one or more such risk factors during the subsequent 12 months follow-up. Notably, prevalence of risk factors was higher in patients with moderate or severe CHE (ie, those patients most likely to receive systemic therapy) and increased with older age.

In recent years, oral JAKis have been approved for several inflammatory diseases, including rheumatoid arthritis, AD, and recently also alopecia areata. Initial safety concerns were raised more than a decade ago and confirmed in the postauthorization safety study, ORALSURV,^15^ which demonstrated increased cardiovascular and cancer risk in patients with rheumatoid arthritis treated with the oral JAKi tofacitinib. Following black box warnings on oral JAKis including tofacitinib, abrocitinib, baricitinib, and upadacitinib, EMA imposed restrictions on the use of oral JAKi in selected patient groups, including subjects ≥65 years of age and people with increased risk of cancer or major cardiovascular problems, as well as previous long-term or current smokers.^7^ Previously, one study found that half of patients had risk factors that would potentially impact the suitability of oral JAKi as a treatment option in adults with AD.^8^ Despite a substantial overlap between AD and CHE, as seen in our study, approximately two-thirds of patients with CHE do not have a history of AD. Furthermore, the markedly higher prevalence (81.7%) of risk factors in our study highlights that a differentiated approach is needed in patients with CHE compared with AD. In contrast to AD, CHE is considered to be a more heterogeneous disease, and inflammatory signature may depend on etiology of CHE (eg, allergic and irritant contact dermatitis) as well as phenotype (eg, hyperkeratotic and vesicular).^16^ Notably, CHE displays a considerable heterogeneity in the inflammatory response, with upregulation of both Th1 and Th2, respectively, depending on CHE subtype.^16^ Interestingly, hyperkeratotic CHE has shown to display a non-AD-like inflammatory footprint with upregulation of type 1 inflammation as well as tumor necrosis factor.^17^

In treating this highly heterogenous disease where a range of cytokines may be upregulated, a broad-acting inhibition of the JAK/STAT pathway could have an important role in achieving sufficient therapeutic response. However, given the established safety concerns with oral JAKi, together with the considerable prevalence of risk factors impacting their use in CHE patients, systemic exposure to JAKi may not be an optimal solution in this population from a safety perspective. Notably, even among patients without apparent risk factors at baseline, a considerable proportion of these patients develop such risk factors over time, further complicating prescription of oral JAKi in this patient population. However, while oral alitretinoin is currently the only systemic treatment approved for severe CHE in Europe and Canada, topical JAKi such as, eg, delgocitinib, which displays negligible systemic absorption, may be a suitable treatment option for patients with moderate-to-severe CHE.^18^

### Strengths and limitations

The current study was conducted using Danish survey data and national registries of routinely collected health care data. The data sources are of high quality and allowed for accurate time-dependent information regarding patients’ risk factors for oral JAKi. However, certain limitations also warrant mentioning. In a global perspective, the Danish population may represent a population with a rather low risk profile compared with some other regions. Estimations of obesity rates in Danish adults (21.3%) are lower than, eg, Germany (25.7%), the UK (29.5%), and the US (37.3%). Furthermore, the prevalence of adults who smoke in Denmark is lower (17.5%) compared with worldwide estimates (23%).^19^, ^20^, ^21^ CHE patients’ risk profiles may therefore not be directly extrapolated in other populations and geographical regions, and it is likely that the prevalence of risk factors is even higher in other countries.

## Conclusion

Approximately eight out of ten patients with CHE in a Danish prospective cohort had at least one risk factor limiting the use of oral JAKi. Among patients without risk factors at baseline, 20% of patients had developed one or more risk factors at 12 months follow-up. While the JAK/STAT pathway may be a favorable therapeutic target for CHE, health care providers should assess risk factors in their patients when choosing treatment for CHE.

## Conflicts of interest

Outside of the submitted work, Dr Egeberg has received research funding from Almirall, Pfizer, Eli Lilly, Novartis, Bristol-Myers Squibb, AbbVie, Janssen Pharmaceuticals, Boehringer Ingelheim, the Danish National Psoriasis Foundation, the Simon Spies Foundation, and the Kgl Hofbundtmager Aage Bang Foundation. He has received honoraria as consultant and/or speaker from Amgen, AbbVie, Almirall, Leo Pharma, Zuellig Pharma Ltd, Galápagos NV, Sun Pharmaceuticals, Samsung Bioepis Co, Ltd, Pfizer, Eli Lilly and Company, Novartis, Union Therapeutics, Galderma, Dermavant, UCB, Mylan, Bristol-Myers Squibb, McNeil Consumer Healthcare, Horizon Therapeutics, Boehringer Ingelheim, and Janssen Pharmaceuticals. He is currently employed by LEO Pharma. Dr Andersen has received speaker honoraria from Eli Lilly Company. Drs Sieborg and Nymand have nothing to declare. Outside of the submitted work, Dr Thomsen has been a speaker or advisor for Sanofi, AbbVie, LEO Pharma, Pfizer, Eli Lilly, Novartis, UCB Pharma, Symphogen, UNION Therapeutics, Almirall, and Janssen Pharmaceuticals and received research support from Sanofi, AbbVie, LEO Pharma, Novartis, UCB Pharma, and Janssen Pharmaceuticals. Outside of the submitted work, Dr Thyssen has been an advisor for AbbVie, Almirall, Arena Pharmaceuticals, Coloplast, OM Pharma, Aslan Pharmaceuticals, Union Therapeutics, Eli Lilly & Co, LEO Pharma, Pfizer, Regeneron, and Sanofi-Genzyme. He has previously received speaker honoraria from AbbVie, Almirall, Eli Lilly & Co, LEO Pharma, Pfizer, Regeneron, and Sanofi-Genzyme, and has received research grants from Pfizer, Regeneron, and Sanofi-Genzyme. He is currently employed by LEO Pharma. Outside of the submitted work, Dr Torres has been investigator, consultant, and/or speaker: AbbVie, Amgen/Celgene, Almirall, Arena, BIOCAD, Biogen, Boehringer Ingelheim, Bristol Myers Squibb, Fresenius-Kabi, Janssen, Leo Pharma, Lilly, Merck, Mylan, Novartis, Pfizer, Samsung-Bioepis, Sandoz, Sanofi Genzyme, and UCB. Outside of the submitted work, Dr Chiricozzi has served as an advisory board member and consultant and has received fees and speaker's honoraria or has participated in clinical trials for AbbVie, Almirall, Bristol Myers Squibb, Boehringer-Ingelheim, Galderma, Leo Pharma, Lilly, Janssen, Novartis, Sanofi Genzyme, and UCB Pharma.
